# The Utility of Virtual Reality in Orthopedic Surgical Training

**DOI:** 10.1016/j.jsurg.2022.06.007

**Published:** 2022-07-09

**Authors:** Nicolas Cevallos, Brian Zukotynski, Danielle Greig, Mauricio Silva, Rachel M. Thompson

**Affiliations:** * UCSF School of Medicine, San Francisco, California; † Department of Orthopedic Surgery, David Geffen School of Medicine at UCLA, Los Angeles, California; ‡ Orthopaedic Institute for Children, Los Angeles, California

**Keywords:** virtual reality, orthopaedic education, coaching, training, Competencies, Medical Knowledge, Learning and Improvement, Systems-Based Practice, Patient Care, Medical Knowledge, Practice-Based Learning and Improvement

## Abstract

**OBJECTIVE:**

To examine the efficacy of virtual reality (VR) to prepare surgical trainees for a pediatric orthopedic surgery procedure: pinning of a slipped capital femoral epiphysis (SCFE).

**DESIGN:**

Participants were randomly assigned to a standard, study guide (SG) group or to a VR training group. All participants were provided a technique video and SG; the VR group additionally trained via an Osso VR surgical trainer (ossovr.com) with real-time feedback and coaching from an attending pediatric orthopedic surgeon. Following training, participants performed a SCFE guidewire placement on a SawBones model embedded in a soft-tissue envelope (SawBones model 1161). Participants were asked to achieve “ideal placement” based on the training provided. Participants were evaluated on time, number of pin “in-and outs,” penetration of the articular surface, angle between the pin and the physis, distance from pin tip to subchondral bone and distance from the center-center point of the epiphysis.

**SETTING:**

Orthopedic Institute for Children, Los Angeles, CA.

**PARTICIPANTS:**

Twenty fourth-year medical students, first- and second-year orthopedic residents without experience with the SCFE procedure.

**RESULTS:**

Twenty participants were randomized to SG (*n* = 10) or VR (*n* = 10). Average time to final pin placement was 19% shorter in VR group (706 vs 573 seconds, p = 0.26). When compared to SG, the VR group had, on average, 70% less pin in-and-outs (1.7 vs 0.5, p = 0.28), 50% less articular surface penetrations (0.4 vs 0.2, p = 0.36), and 18% smaller distance from pin tip to subchondral bone on lateral view (7.1 vs 5.8 mm, p = 0.42). Moreover, the VR group had a lower average angle deviation between pin and line perpendicular to the physis on coronal view (4.9° vs 2.5°, p < 0.05).

**CONCLUSIONS:**

VR training is potentially more effective than traditional preparatory methods. This pilot study suggests that VR training may be a viable surgical training tool, which may alleviate constraints of time, money, and safety concerns with resultant broad applicability for surgical education.

## INTRODUCTION

Advances in surgical techniques, work-hour restrictions, and increased time spent on non-clinical administrative tasks have placed increased demands on surgical trainees.^1,2^ Moreover, a greater emphasis on patient safety along with higher expectations for surgical outcomes and a litigious medical practice atmosphere have placed increased pressure on attending surgeons to be mindful of trainees’ potential missteps.^3,4^ The length of surgical residency, however, has remained fixed, leaving surgical trainees and resident educators in search for safer, more efficient modalities for learning and practicing surgical techniques.^5,6^ Virtual reality (VR) simulation has emerged as a promising alternative. VR simulation provides access to unlimited, safe technical repetitions, enabling the acquisition of surgical skills in a flexible, low-stakes training environment. Training with VR simulators has previously been shown to improve surgeon performance with good transferability to the operating room.^7^

VR surgical training is specifically useful for learning the steps of complex orthopedic procedures requiring multiple tools.^6^ However, it is not clear from the current literature whether VR training is similarly useful in acquiring the basics of accurate pin/screw placement requisite for many orthopedic procedures. The importance of accurate percutaneous pin placement is especially true in pinning slipped capital femoral epiphysis (SCFE). Optimal screw placement is the key to preventing further slippage and hardware-related complications, such as screw cutout or intra-articular penetration.^8–10^ To successfully pin a SCFE, surgeons must be facile with the use of fluoroscopy and have a keen awareness of spatial relationships and how projected fluoroscopic views correspond to 3-dimensional space to enable proper placement of internal fixation and reduce ionizing radiation exposure to the patient, themselves and the operating room staff.

VR simulation is seemingly well-suited to train this skill as programmed VR environments can closely replicate the experience of using 2-dimensional fluoroscopic images to visualize 3-dimensional placement of internal fixation devices. Theoretically, the benefits realized utilizing VR training to familiarize a trainee with the steps of a more complex procedure may extend to more fundamental skill acquisition, with broad implications for multiple orthopedic subspecialties and procedures. Further, VR simulation has the benefit of being able to provide standardized, objective grading of operative parameters including accuracy of surgical technique, economy of motion, and procedural time, making it well-suited for basic skill acquisition.

Therefore, the aim of this study was to compare the performance of novice surgical trainees who trained utilizing VR simulation to those who did not have access to VR training and only studied a standard technique guide (SG) prior to in situ pinning of a SawBones model for SCFE. We hypothesize that adding VR simulation training in preparation for SCFE in-situ pinning will improve performance with broad implications for teaching all other orthopedic procedures.

## METHODS

After receiving Institutional Review Board approval, fourth-year medical students and first- and second-year orthopaedic residents without prior experience of performing a SCFE procedure were recruited and randomly assigned to either the SG or VR group. All participants completed informed written consents. All components of the VR system were provided by Osso VR (http://www.ossovr.com/).

### Study Design

All participants received a sealed envelope randomly assigning them to either the SG or VR groups. All participants received a grading rubric in addition to a written technique guide specific to SCFE pinning and a 6-minute video demonstrating the technical details of a SCFE pinning utilizing a SawBones model. These resources simulate resources readily available to surgical learners preoperatively.

Participants in the SG group had 2 hours to review the surgical technique guide and the demonstration video, which included step-by step instructions and illustrations of the procedure. SG participants did not have access to the VR trainer, nor did they receive any coaching/training with pediatric orthopedic staff prior to SawBones testing.

The participants randomized to VR were allowed to review the study guide and demonstration video prior to VR training. Thereafter, these participants completed brief tutorials on how to properly use the OSSO VR system. The Osso VR system (Osso VR, Sacramento, California) is composed of an Oculus Rift virtual headset that attaches over the eyes and 2 oculus wrist and touch motion controllers that communicate electronically with the headset. The wrist and touch controllers relay vibrations and forces back to the user in the form of haptic, providing tactile as well as visual feedback. The VR system did not undergo any changes or upgrades throughout the duration of the study.

VR participants also completed a specific tutorial on the utilization on the Osso VR SCFE-specific module (SCFE Focused with C-Arm Beta). In the VR environment, participants initially performed an in situ pinning of a SCFE utilizing the system’s training mode, with written instructions and prompts for each step of the procedure provided by the system. This training mode provides immediate feedback to the trainee, grading their pinning accuracy as a product of the angle and depth of final pin placement in both sagittal and coronal views. Grades from A to F (from excellent to poor) were provided. The participants were given up to 2 hours of unrestricted access to the training environment. After the VR participants successfully achieved 3 grade “A” pin placements, they were transitioned into a testing mode with real-time feedback and coaching from an attending pediatric orthopedic surgeon (RT, MS). VR training was completed once subjects successfully completed two additional SCFE pinnings deemed acceptable by the coaching attending surgeon.

**FIGURE A1.**
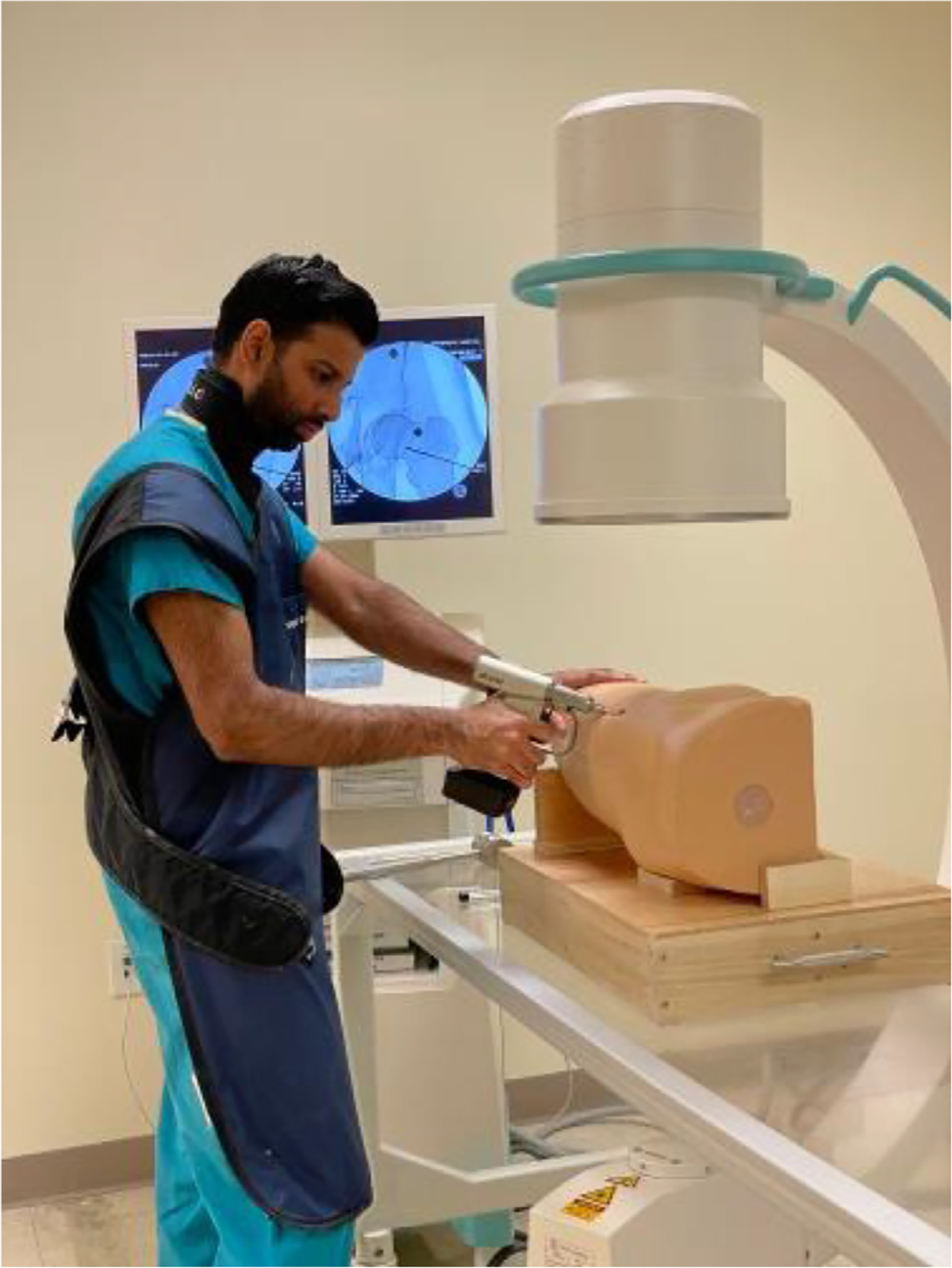
Set-up of the SCFE guidewire placement on a SawBones model embedded in a soft-tissue envelope (SawBones model 1161).

After completion of studying the study guide/technique video (SG and VR groups), and following VR training (VR group), participants were then escorted to a separate test room, where they were tasked with placing a guidewire in a SawBones SCFE model utilizing fluoroscopic guidance. The surgical model consisted of a realistic artificial proximal femur of a moderate SCFE (SawBones model 1161), embedded in a ballistic gel thigh model to simulate the soft tissue envelope of the hip. The artificial bones were radio-opaque to allow fluoroscopic-guidance of pin placement (Fig. A1). The “surgical incision” was pre-made into the soft tissue envelope prior to the exercise, as the goal of this study was to focus on pin placement rather than surgical dissection and approach. The same soft tissue envelope was used for each participant, enabling standardization of the surgical incision. A new SawBones femur was used for each study participant.

All participants received instruction on proper use of drill instruments by an attending surgeon prior to testing. Subjects had up to 20 minutes to perform the procedure and were given an unlimited number of pinning attempts and unrestricted use of fluoroscopy to complete the task. The procedure was timed. The time started once the participant indicated that they were ready to start and ended once the participant indicated that they were satisfied with the final position of the pin or once the 20-minute time limit was reached. Each participant was video recorded for later in-depth analysis.

### Evaluation of SCFE Procedure

Data collection was performed at the time of the exercise and later confirmed with the video recordings. Data collectors were blinded to the training modality given to the participant, and all data was free of personal identifiers. Data collection included the following: total time-to-pin-placement, number of “in-and-outs” (pin in-and-out of bone), number of fluoroscopy images taken, whether or not the pin penetrated the articular surface of the femoral head (assessed both radiographically and via the physical model), angle between the pin and the physis, and pin-tip location within the femoral head, measured as distance from the tip of the pin to the center-center point (ideal pin location) and pin distance from the subchondral bone (in mm). Scoring rubrics based on these criteria were provided to all participants prior to testing (Table A1).

Mann-Whitney-Wilcoxon tests were utilized to compare continuous variables with statistical significance set at p < 0.05. All statistical analysis was conducted using GraphPad Prism Statistics/ Data Analysis software (GraphPad Software, Inc., La Jolla, CA).

## RESULTS

**FIGURE 1.**
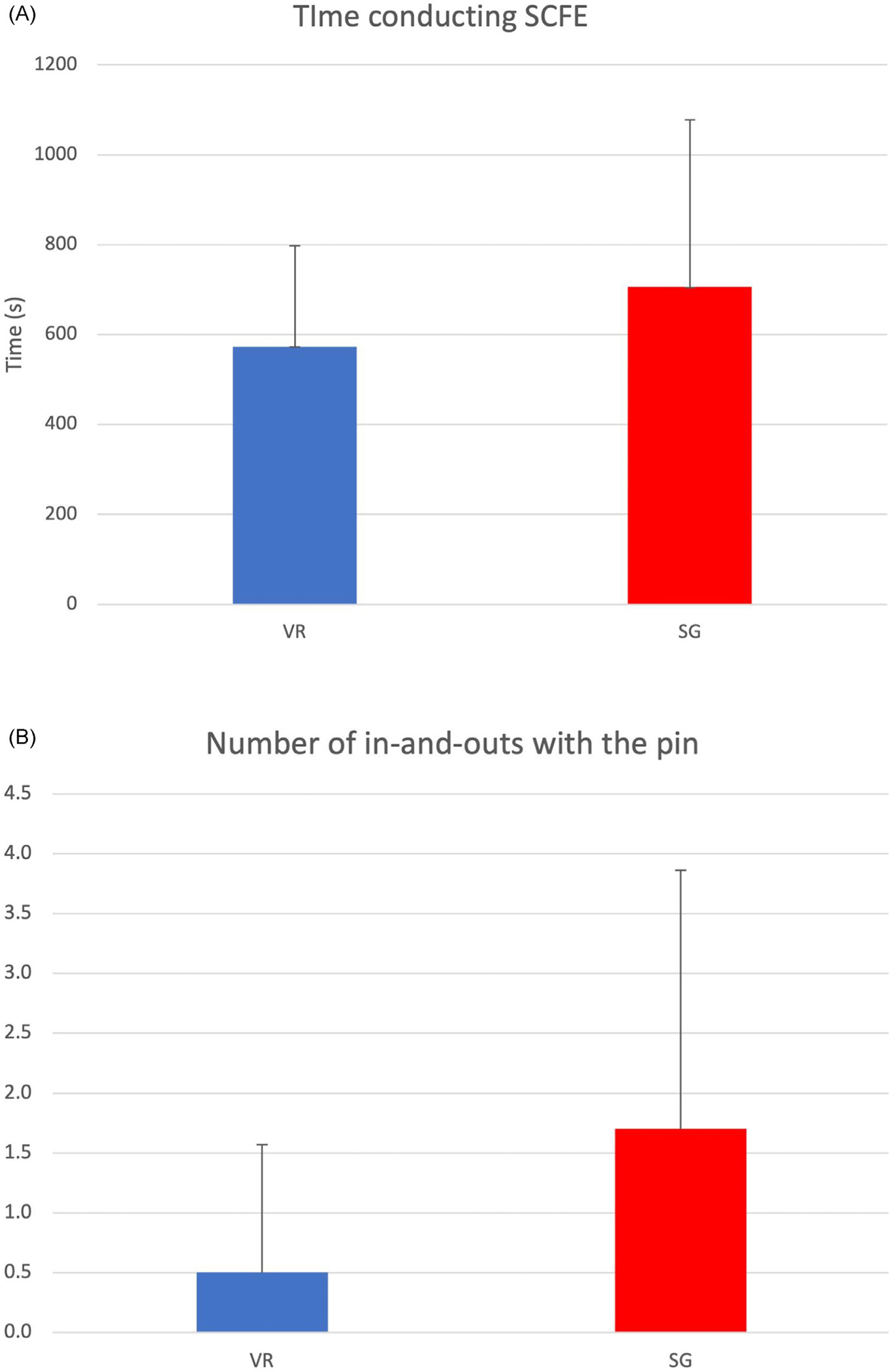

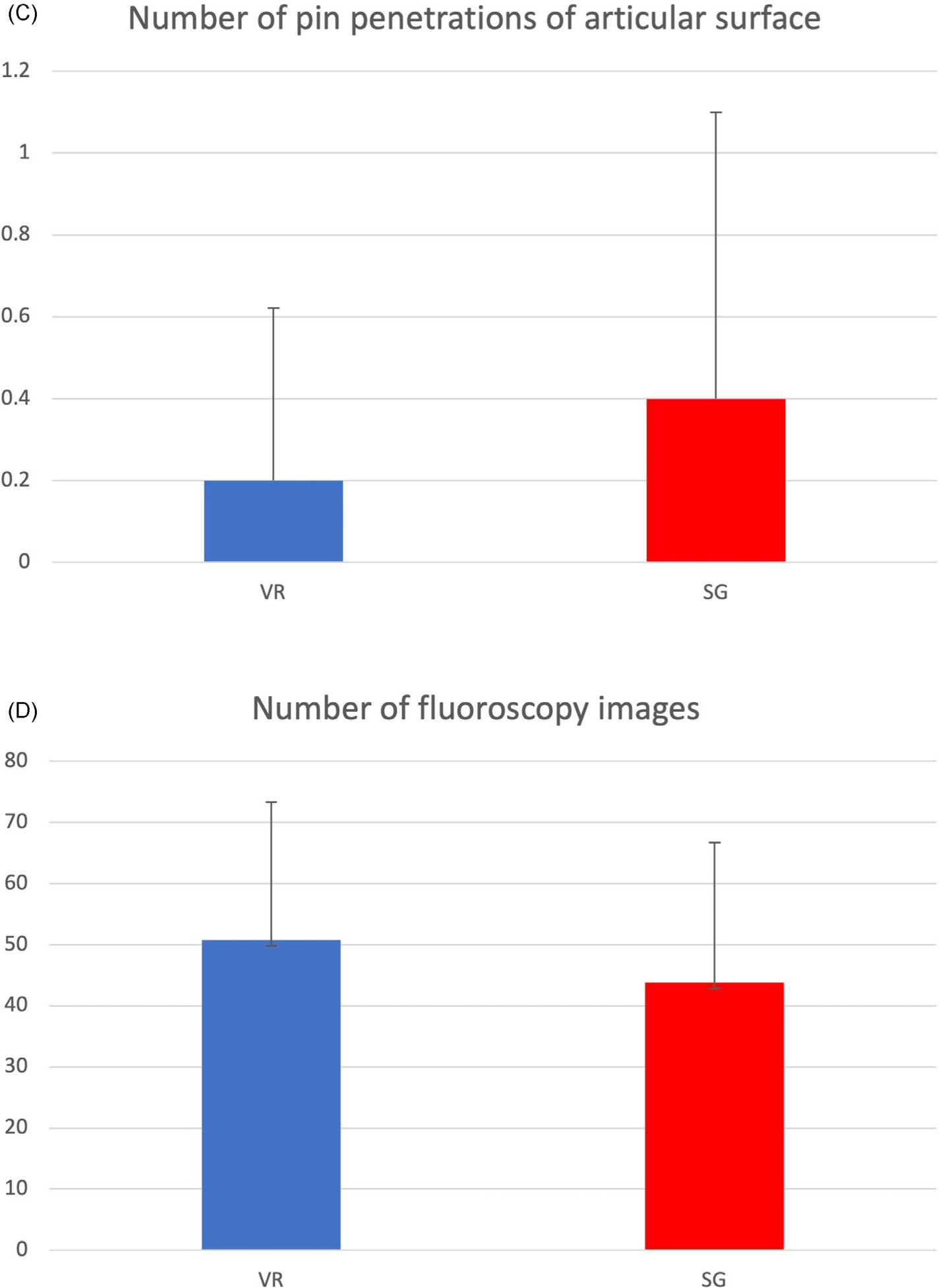
(A) Time to complete SCFE pinning. Statistical analysis was performed using Mann-Whitney-Wilcoxon test. Differences within each group were not statistically significant. (B) Number of in-and-outs with the pin. Statistical analysis was performed using Mann-Whitney-Wilcoxon test. Differences within each group were not statistically significant. (C) Number of pin penetrations of articular surface. Statistical analysis was performed using Mann-Whitney-Wilcoxon test. Differences within each group were not statistically significant. (D) Number of fluoroscopy images. Statistical analysis was performed using Mann-Whitney-Wilcoxon test. Differences within each group were not statistically significant.

Twenty participants were recruited, including 9 senior medical students, 6 first-year surgical residents, and 5 second-year surgical residents; 10 were randomized to the SG group and 10 to the VR group (Table 1). On average, participants in the VR group completed the SawBones assessment 132.60 seconds faster than the SG group (SG: 705.70, VR: 573.10; p = 0.26; Fig. 1A). The VR group had 70% less pin “in-and-outs” (SG: 1.7, VR: 0.5, p = 0.26; Fig. 1B) and 50% less articular surface penetrations (SG: 0.2, VR: 0.4; p = 0.36; Fig. 1C). However, subjects in the SG group utilized less fluoroscopic images compared to VR (SG: 43.80, VR: 50.80; p = 0.19; Fig. 1D).

With regards to pin placement, results were variable. In the coronal plane, the VR group had a lower average angle deviation between the pin and a line perpendicular to the physis (SG: 4.90°, VR: 2.55°, p < 0.05; Table 2). However, the SG group demonstrated a lower average angle deviation between the pin and a line perpendicular to the physis in the sagittal plane (SG: 4.95°, VR: 5.70°, p = 0.43; Table 2), although this result did not reach statistical significance. Analysis of pin location with respect to center-center position revealed that the SG group was closer in both the coronal (SG: 4.86 mm, VR: 6.51 mm, p = 0.46; Table 3) and sagittal (SG: 8.22 mm, VR: 8.81 mm, p = 0.46; Table 3) planes. As for pin location with respect to distance from the subchondral bone, on average, the SG group was closer than the VR group in the coronal plane (SG: 5.83 mm, VR: 7.23 mm, p = 0.46; Table 4), but the VR group was closer in the sagittal plane (SG: 7.14 mm, VR: 5.79 mm, p = 0.42; Table 4).

**FIGURE 2.**
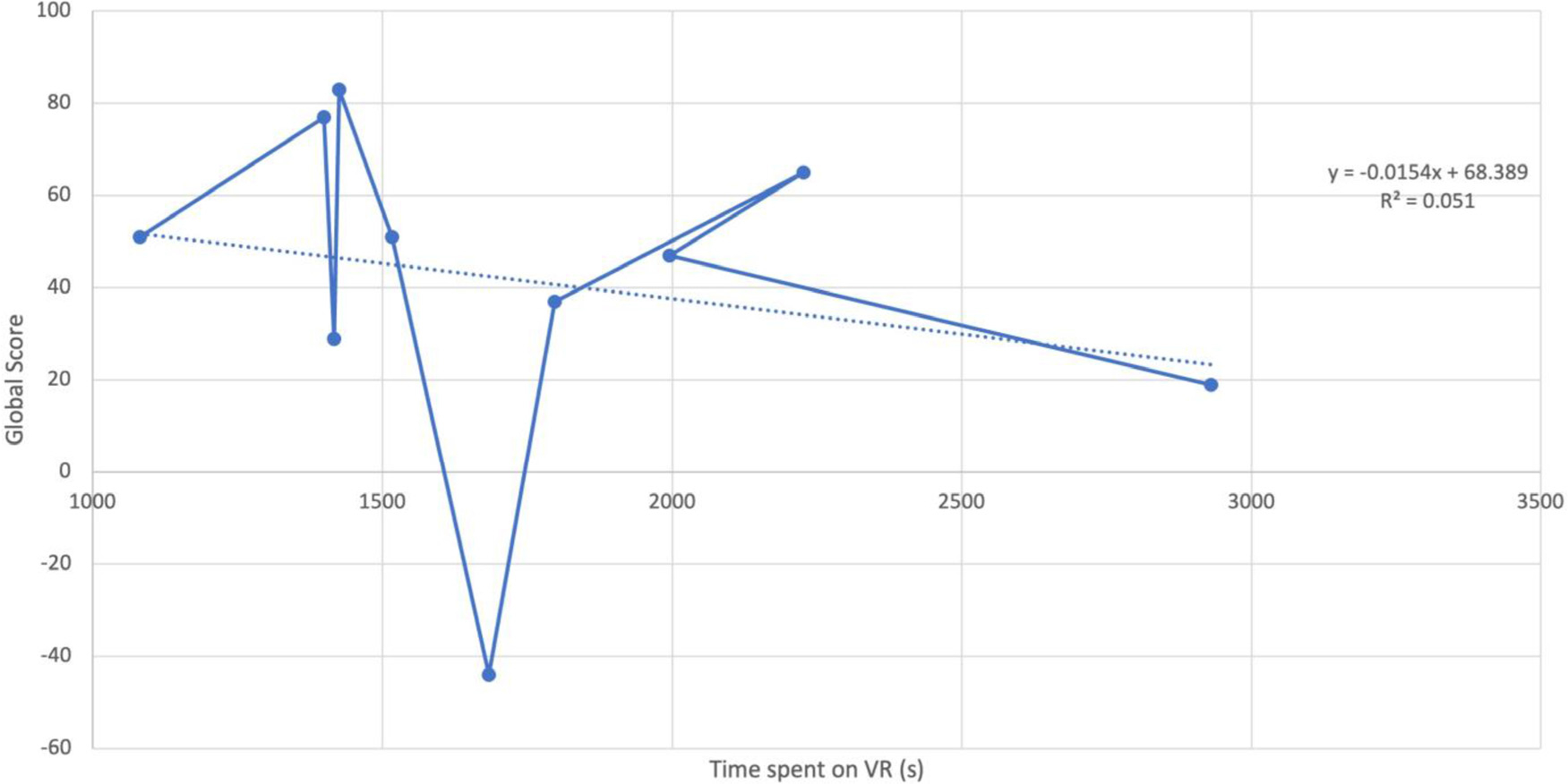
Correlation of time spent on VR training and aggregate score. Individuals who successfully completed the VR module faster achieved a higher global score compared to individuals who were slow to complete the VR module (R^2^ = 0.05).

In a subgroup analysis of the VR participants, we found that time-to-complete-VR training correlated with an overall global surgical score (score calculated to incorporate results from all tested parameters). Individuals who successfully completed the VR module faster achieved a higher global score compared to individuals who were slow to complete the VR module (R^2^ = 0.05) (Fig. 2).

## DISCUSSION

The purpose of this study was to evaluate whether VR surgical training better prepares surgical trainees for common operative procedures compared to traditional methods of preparation (written surgical technique guide and demonstration video). Our findings indicate that VR training trended toward improved skill acquisition and application in preparation for SCFE pinning, with implications for improved acquisition of general orthopaedic skills (i.e., use of fluoroscopy, spatial awareness). Although limited by the number of subjects, the current study suggest that VR training appears to be more effective than traditional preparatory methods with respect to achieving a shorter procedure time, decreasing the number of “in-and-out” events, decreasing the number of violations of the joint space, and achieving a better overall pin placement.

Further, given the correlation between VR training time and outcomes, VR training and testing may help identify resident baseline surgical skill level and provide opportunities for additional, individualized VR training in those trainees whose baseline skill set is lower than their peers. In this light, VR surgical trainers may prove beneficial as a much-needed objective assessment tool that can measure trainee progression, allowing for standardization of training and testing, ensuring that all surgical trainees graduate with a similarly tuned skill set.

Moreover, VR provides a safe, interactive learning environment that provides a necessary adjuvant to more traditional passive lecture-based learning, which remains common in surgical training programs. Active learning methods, including VR simulation, improve acquisition of knowledge, increase motivation for self-directed learning and also increase the transfer of skills to clinical practice.^11–13^ Previous research demonstrates VR simulation’s success in training bronchoscopy, laparoscopic skills, and operating room performance.^14–16^ Specifically as VR relates to orthopaedics, Blumstein et al. demonstrated the utility of VR training in a tibial nail SawBones model. These authors found that compared to those without VR training, individuals who trained utilizing VR simulation prior to tibial nailing completed a significantly higher number of steps correctly, completed the module quicker, and had a higher knowledge for instruments.^6^ However, this study primarily analyzed steps completed, demonstrating the utility of VR training for familiarizing oneself with the steps of a procedure. On the contrary, this present study demonstrates the utility of VR training for technical skill acquisition and finesse. Similarly, Lohre et al. demonstrated the superiority of VR simulation for technical skill acquisition and implant placement.^17^ Our study corroborates these findings and expands them to include findings supporting the use of VR training for tactile feedback, spatial awareness with the use of fluoroscopy.

Additionally, our study demonstrated the viability of combining VR training with live surgical coaching, which allowed our coaches to individualize guidance, tailoring each trainees’ experience to their skills and perceived deficits. And while we incorporated live coaches, available software allows for remote coaching/feedback, greatly expanding the potential use of VR training globally. Moreover, as stated previously, VR training may identify learners who would benefit from more pre-OR training. Coupling coaching with VR training would allow for this training to be completed in a safe, cost-effective environment, without OR time constraints, risks to patients or the expenses associated with SawBones and cadavers.^18^ This combination may serve to “level the playing field” in surgical training, ensuring a baseline level of competence, safety, and familiarity with the procedure prior to entering the operating room. Such assurances may allow for earlier entrustment and autonomy in the OR for more trainees. In fact, increased entrustment, equitable advancement of autonomy, and resultant acceleration in surgical skill acquisition has been previously tied to surgical coaching.^19,20^ And coupling coaching with VR simulation allows for a cost- and time-effective modality by which resident educators may incorporate coaching into successful surgical training programs.

Utilizing VR training and testing for SCFE pinning and similar procedures and concepts may help identify which residents are ready for surgical autonomy. In this way, VR may decrease the impact of implicit bias inherent in choosing which trainee(s) are capable of independence. Such implicit biases are more likely to occur with female and underrepresented residents compared to their white male counterparts.^21–23^ Such biases can lead to slower progression toward autonomy, less surgical exposure, and decreased trainee confidence. In fact, a recent systematic review found that while there were no differences in performance or skills between men and women at any level of training in medicine, women rated themselves lower in perceived clinical skills, performance, confidence in procedures, identification with the role of doctor, interpersonal/communication skills, and preparedness for leadership positions.^24^ In this light, VR training systems with objective assessment capability can serve as an invaluable tool to allow faculty to evaluate resident performance without bias and allow for graduated autonomy when objectively appropriate.

## LIMITATIONS AND CONCLUSION

Despite the promising results, this study had several limitations, including the inclusion of fourth-year medical students as opposed to solely first- and second-year orthopedic surgery residents. However, the level of surgical training between a second-year resident, first-year resident, and fourth-year medical students is similar considering the timing of when our study occurred (July-September). Second year residents were newly transitioning to orthopedics from their intern year positions, which primarily consisted of administrative and patient management duties. Moreover, the lack of surgical experience amongst the more junior participants was advantageous in that it allowed for an accurate measure of the differential between typical preparation and VR training unencumbered by previous experience.

Further, the interpretation of the significance of study results was limited by our small sample size. Although the findings in the VR group showed promising results, they were not statistically significant differences due to the variability in baseline skills and performance amongst participants. Given the limited sample size, the pin-physeal angle was the only statistically significant improvement noted in the VR group. While the argument could be made that this is not clinically relevant as there is emerging evidence that screw angle does not necessarily affect stability,^25^ the accepted standard for screw placement remains perpendicular to the physis.^26^ As such, residents were instructed on perpendicular placement, and individuals who completed VR training demonstrated significantly increased accuracy, a highly transferable skill. That being said, future experiments will include multiple institutions to obtain a larger sample size. Longitudinal studies evaluating the effectiveness of utilizing VR for individualized training plans would also be of benefit.

Overall, our pilot study demonstrates that VR training is a potentially effective novel surgical training tool, which may alleviate the constraints of time, money, and safety concerns on surgical education. While further investigation with a larger number of subjects is warranted, VR appears to be a promising, efficacious, and efficient educational tool that warrants consideration as an adjunct to traditional orthopedic resident education.

## Figures and Tables

**TABLE 1.** Demographic Characteristics of Included Study Participants

Characteristic	VR (*n* = 10)	Study Guide (*n* = 10)

Gender		
Male	6	8
Female	4	2
Level of training		
Medical student-4	4	5
PGY-1	4	2
PGY-2	2	3

**TABLE 2.** Angle Between the Pin and the Physis

	VR Coronal Plane Angle Between the Pin and the Physis	SG Coronal Plane Angle Between the Pin and the Physis	VR Sagittal Plane Angle Between the Pin and the Physis	SG Sagittal Plane Angle Between the Pin and the Physis

Average	2.55	4.90	5.70	4.95
SD	1.42	3.00	4.06	3.13
Total score	25.5	49.00	57.00	49.50
p Value	0.02		0.43	

**TABLE 3.** Pin Location with Respect to Center-center Position

	VR Coronal Plane Pin Location with Respect to Center-Center Position	SG Coronal Plane Pin Location with Respect to Center-Center Position	VR Sagittal Plane Pin Location with Respect to Center-Center Position	SG Sagittal Plane Pin Location with Respect to Center-Center Position

Average	6.51	4.86	8.81	8.22
SD	5.47	2.15	6.37	5.06
Total score	65.08	48.56	88.07	82.17
p Value	0.46		0.43	

**TABLE 4.** Pin Location with Respect to Distance from the Subchondral Bone

	VR Coronal Plane Pin Location with Respect to Distance from the Subchondral Bone (Goal = 5 mm or Less)	SG Coronal Plane Pin Location with Respect to Distance from the Subchondral Bone (Goal = 5 mm or Less)	VR Sagittal Plane Pin Location with Respect to Distance from the Subchondral Bone (Goal = 5 mm or Less)	SG Sagittal Plane Pin Location with Respect to Distance from the Subchondral Bone (Goal = 5 mm or Less)

Average	7.23	5.83	5.79	7.14
SD	6.50	3.36	3.93	6.16
Total score	72.32	58.26	57.88	71.38
p Value	0.49		0.42	

## Appendix

**TABLE A1.** Participants were Provided with this Chart Prior to the Test and were Evaluated After Completion of the SCFE Pinning

*Time to place your final pin*
1 point rewarded for each minute *under* 10 min
*Number of in-and-outs with the pin*
1 point removed for each in-out-in *over* 5
*Number of fluoroscopy images taken*
1 point removed for each image *over* 10
*Whether or not the pin penetrated the articular surface*
1 point removed each time the pin penetrates the articular surface (on fluoroscopy or final construct)
*Angle between the pin and the physis*
1 point removed each degree off of perpendicular on both AP and lateral views (measured on x-ray)
*Pin location with respect to center-center position on AP and lateral hip views*
1 point removed for each 1 mm distance from center-center in any direction (measured on SawBone)
*Pin location with respect to distance from the subchondral bone (goal = 5 mm or less)*
1 point removed for each 1 mm additional distance from 5 mm (measured on x-ray)
